# News and Coming Events

**Published:** 1907-11-30

**Authors:** 


					NEWS AND COMING EVENTS.
H.R.H. Princess Christian of Schleswig-Holstein
will open the new operating theatres at the Royal Free
Hospital, Gray's Inn Road, on Tuesday afternoon next at
3.30 r.M.
A reception will he held at 33 Devonshire Street, Harley
Street, W., on Wednesday evening, December 4 next, from,
:9 to 11 r.M., by Sir William Bennett, K.C.Y.O., on behalf of
.the Incorporated Institute of Hygiene.
On Monday last an At Home was given by the President
and Committee of the Royal Southern Hospital, Caryl
Street, Liverpool. The wards and various departments of
the hospital were open for inspection, and a demonstration
of the uses of x-rays was given by Dr. David Morgan.
Mr. Rickman J. Godlee will deliver the Bradshaw
lecture at the Royal College of Surgeons on Friday, Decem-
ber 6 next, at 5 o'clock r.M. The subject of the lecture
will be " The Prognosis and Treatment of Tubercular
Disease of the Genito-Urinary Organs."
Lady Lttdlow will visit St. Bartholomew's Hosoi-
Thursday, December 5, at 2.30 r.M., fo?'*'1 Lne Pul'Pose
laying the foundation-stone of the nei'i " Pathological bloc'-
HIS'
The inaugural dinner of the Ro Society of Medicin
take place at the Hotel Cecil o ' n Tuesday, December 3
TUP
at 7.30 r.M 1110
A Conference, to discuss thite 6 <lueftion of estaoiisbi** >
out-patient department of a ,, ,restricted and consul
character in connection with f,J 11116 fronts' Hospital, ^1
held in the Lecture Theatre ' 1 of tlle Hospital, on w
November 29, at 4.30 p.m., Dl0vr' ^ Allan, Medical ? \
of Health for Westminster,J 1in t^le c^aii\
nr
The usual spring cours<na'3 ?f Posfc~graduate lectures a
Berlin University commejj nces on March 2 next. All <>e
of the course and particu'rT .iars as to ^ees> etc-> may ke ?^J
on application to Herr T^^e^zer, Langenbeck Hause, Zlj
strasse 10, Berlin.

				

